# NGAL as a Biomarker of Tubular Injury and Glomerular Dysfunction in Fabry Nephropathy

**DOI:** 10.3390/cells15191797

**Published:** 2026-10-01

**Authors:** Margarita M. Ivanova, Julia Dao, Leah Svarny, Emily Nguyen, Sahithi Ponnam, Ozlem Goker-Alpan

**Affiliations:** Lysosomal and Rare Disorders Research and Treatment Center, Fairfax, VA 22030, USAogoker-alpan@ldrtc.org (O.G.-A.)

**Keywords:** Fabry disease, NGAL, kidney, nephropathy, eGFR, podocytes, tubular, Gb-3, lyso-Gb-3

## Abstract

**Highlights:**

**What are the main findings?**

- Plasma and urinary NGAL levels are significantly elevated in Fabry disease patients, even with preserved eGFR, indicating subclinical tubular injury.
- NGAL shows sex-dependent patterns: urinary NGAL is elevated across all stages in both sexes, while plasma NGAL correlates strongly with declining eGFR, particularly in male patients.

**What are the implications of the main findings?**

- Urinary NGAL serves as a sensitive biomarker for early detection of tubular damage in Fabry nephropathy, offering added value over conventional markers such as eGFR.
- NGAL measurement may improve risk stratification, timely therapeutic intervention, and monitoring of treatment response in patients with Fabry disease.

**Abstract:**

Fabry disease (FD) is an X-linked lysosomal disorder caused by pathogenic variants in the GLA gene, resulting in α-galactosidase A deficiency and accumulation of Gb3/lyso-Gb3 (globotriaosylceramide/globotriaosylsphingosine) in cells, including podocytes and renal tubular cells. While podocyte damage drives glomerular dysfunction, tubular dysfunction leads to atrophy and fibrosis, often preceding a detectable decline in glomerular filtration rate (eGFR). Neutrophil gelatinase-associated lipocalin (NGAL) is a marker of renal injury, with urinary and plasma NGAL reflecting distinct aspects of renal dysfunction; however, their clinical value in Fabry nephropathy (FN) remains poorly defined. We explored the clinical relevance of NGAL in distinguishing tubular atrophy from glomerular podocytopathy. Thirty-three FD patients were categorized into groups without and with FN using eGFR. In males, elevated NGAL strongly correlated with FN. In females, urine NGAL was increased regardless of nephropathy status, while plasma NGAL rose mainly in those with reduced eGFR. This pattern suggests that tubular damage and early tubulointerstitial fibrosis (TIF) may precede glomerular impairment in female patients. Conclusions: Tubular damage may precede glomerular dysfunction in FD, and NGAL may serve as a sensitive biomarker of early tubular injury, including before overt nephropathy. NGAL may provide complementary, noninvasive tools for detecting and monitoring renal involvement in Fabry disease.

## 1. Introduction

Fabry disease (FD), also known as Anderson–Fabry disease, is a rare X-linked lysosomal storage disorder caused by mutations in the *GLA* gene, resulting in deficient or absent α-galactosidase A (α-Gal A) enzyme activity. α–Gal A catalyzes the lysosomal hydrolysis of globotriaosylceramide (Gb-3) to lactosylceramide and digalactosylceramide (Gal-Gal-Cer) to galactosylceramide (Gal-Cer) [1,2]. This enzymatic deficiency leads to the progressive accumulation of globotriaosylceramide (Gb3) and its deacylated form, globotriaosylsphingosine (lyso-Gb3), in lysosomes across various cell types, including vascular endothelium, smooth muscle cells, cardiomyocytes, neurons, and renal cells [3]. The disease exhibits a wide phenotypic spectrum, ranging from the classic severe form in hemizygous males to more variable presentations in heterozygous females, affected by factors such as residual enzyme activity and X-chromosome inactivation.

Nephropathy is one of the major contributors to morbidity and mortality in FD. The standard non-invasive clinical laboratory tests for kidney disease (creatinine (Cr), glomerular filtration rate (eGFR), and cystatin C) are not sufficiently sensitive in early stages of Fabry nephropathy (FN) and may delay therapeutic interventions. Moreover, the Fabry-specific biomarkers Gb-3 and Lyso-Gb-3 are not considered clinical entities associated with kidney damage in patients. Therefore, precise detection of the presence and progression of kidney involvement is crucial for preventing late, irreversible complications in FD and for developing effective therapies.

In FD, the typical clinical signs of kidney involvement may be absent, but biopsies may already show glomerular and interstitial damage with early fibrosis. Renal histology may show intracellular Gb-3 deposits in podocytes and epithelial cells, even with normal eGFR and minimal proteinuria. Moreover, heterozygote females develop the same kidney histoinvolvement as hemizygous males with FD, but with a milder spectrum, delayed progression, and a mosaic pattern of Gb3 accumulation due to random X-chromosome inactivation [4,5].

**Figure 1:**
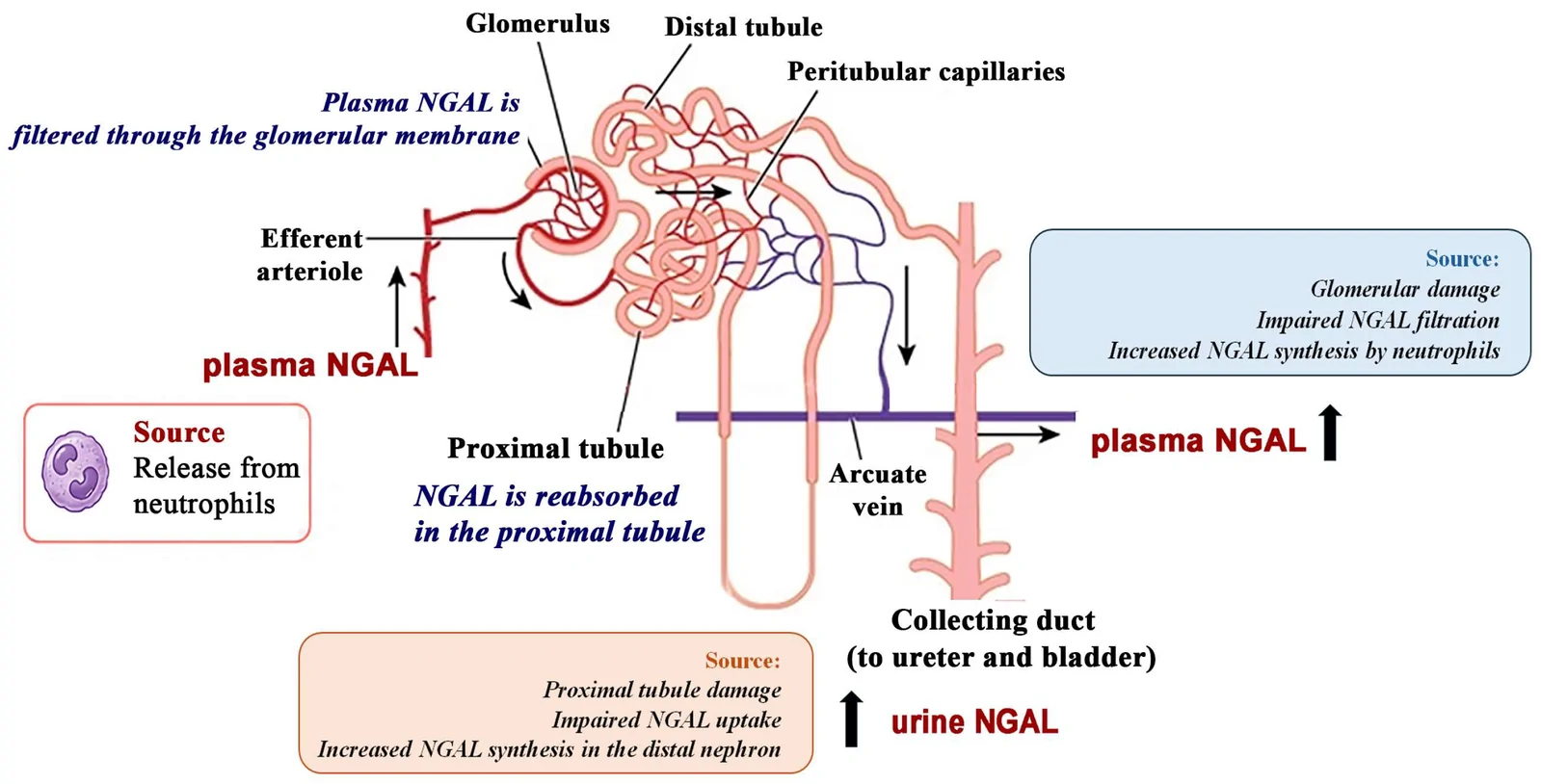
Schematic diagram illustrating the sources of NGAL in the nephron if kidney damage. Plasma NGAL enters via the afferent arteriole and is filtered through the glomerular membrane. In normal conditions, it is reabsorbed in the proximal tubule. Additional sources of NGAL in plasma include release from neutrophils. If glomerular damage occurs, filtration is impaired, leading to increased plasma NGAL levels. If proximal tubular damage occurs, reabsorption is impaired, leading to elevated NGAL in the distal nephron and increased synthesis in the distal tubule. Therefore, NGAL is excreted into the urine via the collecting duct. Elevation of urine NGAL is an indicator of tubular damage.

To improve kidney diagnostics and patient outcomes, highly sensitive, non-invasive biomarkers that capture the cellular specificity of renal pathophysiology are being actively investigated. Functional filtration biomarkers, such as cystatin C, together with tubular injury biomarkers, such as urinary Neutrophil Gelatinase-Associated Lipocalin (NGAL) and kidney injury molecule-1 (KIM-1), are among the biomarkers being evaluated for clinical relevance [6,7,8,9]. NGAL is a secretory protein produced by neutrophils and epithelial cells (including renal tubular cells), detectable in blood and urine, particularly in pathological conditions [10,11]. This biomarker is a promising early-response indicator for acute kidney injury (AKI), chronic kidney disease (CKD), and diabetic nephropathy [10,12,13]. During AKI, NGAL can be detected 2–3 days before a rise in serum creatinine levels, making it the first AKI biomarker to receive U.S. Food and Drug Administration (FDA) clearance for pediatric patients in 2023. While NGAL provides a rapid response to kidney injury during AKI, its response is more complex in chronic inflammation, enabling the prediction of which type of renal tissue is under stress (Figure 1). NGAL is filtered through the glomerular membrane and reabsorbed in the proximal tubule of the kidneys [10]. Elevated NGAL levels in urine suggest proximal tubular damage or might indicate increased synthesis in the distal part of the nephron [14]. Increased plasma NGAL levels can arise from multiple factors, with chronic inflammation playing a significant role. One source of plasma NGAL is the activation of neutrophils and monocytes [15]. In diabetic nephropathy, serum NGAL concentrations rise and correlate with the severity of kidney damage [16]. Additionally, NGAL outperforms traditional markers, such as cystatin C and creatinine, for early detection of CKD and in predicting its progression [17,18]. It is inversely correlated with eGFR, indicating disease severity. Mechanistically, NGAL is secreted by injured tubular cells and modulates apoptosis, while activating the EGFR/HIF-1α signaling pathway (Figure 1) [11]. Correlations between NGAL levels and hemoglobin, iron parameters, and transferrin saturation have also been reported, suggesting that NGAL may serve as a biomarker for assessing iron status in CKD [19]. NGAL levels are elevated across various CKD etiologies and exhibit strong diagnostic and prognostic value in conditions such as IgA nephropathy, systemic lupus erythematosus and lupus nephritis, diabetic nephropathy, and autosomal dominant polycystic kidney disease (ADPKD).

The current study aimed to investigate the clinical value of plasma and urinary NGAL in characterizing tubular and glomerular renal involvement in patients with Fabry nephropathy.

## 2. Materials and Methods

### 2.1. Subjects

In an IRB-approved protocol (Western Institutional Review Board-Copernicus Group Institutional Review Board (WCG IRB) protocol #20233966, ClinicalTrials.gov identifier # NCT06065605), 35 patients with FD (13 males and 22 females) (mean age: 42 ± 14 yrs) consented to the study (Table 1). Two patients were excluded later. Urine from 20 healthy controls (10 males and 10 females) (mean age: 39 ± 11 yrs) was purchased (Innovative Research, Inc., Novi, MI, USA) as single-donor human urine samples. Donor age and sex were specified for each sample. Plasma samples were obtained from healthy donors under protocol NCT02000310 or purchased from Innovative Research, Inc. (Novi, MI, USA).

The FD cohort was stratified by estimated glomerular filtration rate (eGFR) and clinical stage of renal involvement (Table 2). The Stage 1 renal involvement (RI−) group included 9 females and 8 males, whereas the Stage 2–3 renal involvement (RI+) group included 11 females and 5 males. Clinical information was obtained from the patients’ medical records as part of routine clinical assessment. FD diagnosis and genetic confirmation, clinical nephropathy staging, and measurements of plasma and/or urinary Gb3 and lyso-Gb3, eGFR, and cystatin C were extracted from the medical records. eGFR values were obtained from the patients’ medical records, measured by Quest Diagnostics or LabCorp, and calculated using the CKD-EPI equation. Renal involvement was classified according to the clinically determined nephropathy stage and eGFR. Proteinuria data were retrospectively obtained from the patients’ clinical charts. One patient with stage 1 is treatment-Naive, and two patients are “Pseudo-Naive” (Table 1).

### 2.2. Sample Collection and Processing

Urine samples were collected during clinical visits, centrifuged at 2000× *g* for 2 min, immediately aliquoted, and stored at −80 °C until biomarker analysis. When applicable, pregnancy testing was conducted. eGFR, cystatin C, and circulating Gb-3 and lyso-Gb3 were obtained from medical charts, whereas the tests were performed using standard clinical laboratory protocols. Plasma was obtained from venous blood samples collected in EDTA Vacutainer tubes using the NCT02000310 protocol as previously described [20,21]. The blood was centrifuged for 5 min at 5000× *g*, and plasma was collected, aliquoted, and stored at −80 °C before analysis. All samples were collected exclusively for research purposes and assigned unique code IDs to ensure anonymity.

### 2.3. Enzyme-Linked Immunosorbent Assay (ELISA)

As previously described, venous blood samples were collected in EDTA vacutainer tubes. Levels of NGAL were measured in 10 µL of plasma or 25 µL of urine using a commercial Human NGAL (LCN2) ELISA kit with a curve range 31 pg/mL–2000 pg/mL, a sensitivity of 11 pg/mL (EA200004, Origene, Rockville, MD, USA) according to the manufacturer’s instructions. Samples were analyzed in duplicate, and the mean of the duplicate measurements was calculated. Samples were analyzed in at least two independent assay runs. Healthy control and FD samples were analyzed on the same 96-well plate to minimize inter-assay variability.

### 2.4. Statistical Analysis

Statistical analysis was performed using GraphPad Prism version 11 (GraphPad, San Diego, CA, USA). Differences between the two groups were tested using an unpaired Student’s *t*-test with the F-test, and the nonparametric Mann–Whitney U test. Estimation plots were used to visualize differences between groups, along with their 95% confidence intervals. For comparisons involving more than two groups, one-way analysis of variance (ANOVA) or the non-parametric Kruskal–Wallis test was used, as appropriate. The linear correlation between NGAL, age, and clinical biomarkers (eGFR, cystatin-C, plasma Gb3 and Lyso-Gb3, urine Gb3) was assessed using the Multiple Variable Correlation Matrix function in GraphPad Prism to calculate Pearson correlation coefficients, or for pairs of continuous variables, a bivariate analysis was performed using the Pearson correlation coefficient to assess linear relationships. *p* < 0.05 was considered statistically significant. thresholds of uNGAL > 400 and pNGAL > 700 were selected based on levels observed in healthy controls and used to classify tubular and glomerular involvement, respectively. These thresholds were established for analytical purposes and are not clinically validated cutoff values.

## 3. Results

### 3.1. Plasma and Urine NGAL Are Elevated in Patients with FD

**Figure 2:**
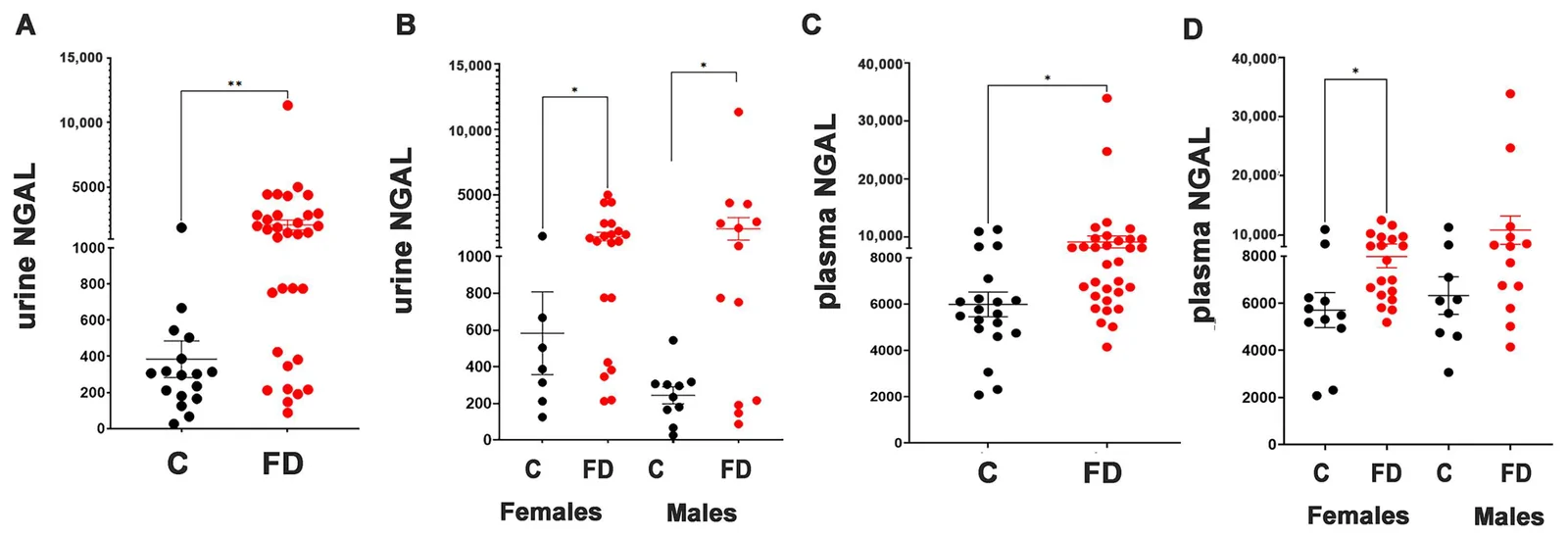
Levels of NGAL. (**A**) Urinary NGAL levels are compared between healthy controls and patients with FD. Statistical analysis using an unpaired *t*-test between the cohorts demonstrated a significant difference between the control and FD cohorts. NGAL (pg/mL) is represented as mean ± SEM, *p* = 0.002, unpaired *T*-test. Nonparametric Mann–Whitney U test, two-tailed, *p* < 0.001. (**B**) The stratification of uNGAL levels by sex showed that patients with FD, both male and female, had significantly higher uNGAL levels than healthy controls (male and female), *p* = 0.02 for females and *p* = 0.002 for males, unpaired *T*-test. Nonparametric Mann–Whitney U test, two-tailed, *p* = 0.016 for females, and *p* = 0.021 for males. (**C**) The plasma NGAL levels in healthy controls vs. patients with FD. Statistical analysis using an unpaired *T*-test to compare the cohorts showed a significant difference between the control and FD groups. Mean ± SEM, *p* = 0.03, *T*-test. Nonparametric Mann–Whitney U test, two-tailed, *p* < 0.0025. (**D**) Plasma NGAL levels in control females and female patients with FD, and control males and male patients with FD. Mean ± SEM, *p* = 0.03 *T*-test. Nonparametric Mann–Whitney U test, two-tailed, *p* = 0.007 for females, and *p* = 0.09 for males. * *p* < 0.05, ** *p* < 0.01.

NGAL is produced by tubular cells and released by the epithelial cells of the thick ascending limb of Henle’s loop and released into the blood and urine (Figure 1) [22]. It has been suggested that uNGAL reflects tubular damage, whereas pNGAL may more indicates glomerular filtration capacity [13,23]. In our cohort, urinary and plasma NGAL levels were significantly higher in patients with FD than in healthy controls (Figure 2A,C). When stratified by sex, the majority of female and male patients had significantly elevated uNGAL levels, suggesting evidence of tubular injury (Figure 2B). Plasma NGAL levels were also significantly increased in FD patients overall (Figure 2C). When stratified by sex, pNGAL levels were significantly elevated in female patients but did not reach statistical significance in male patients, although the two highest pNGAL values were observed in male patients (Figure 2D). These findings suggest sex-dependent differences in NGAL profiles, with female patients showing higher circulating NGAL levels in addition to elevated uNGAL.

### 3.2. NGAL Levels Increased with the Progression of Nephropathy

**Figure 3:**
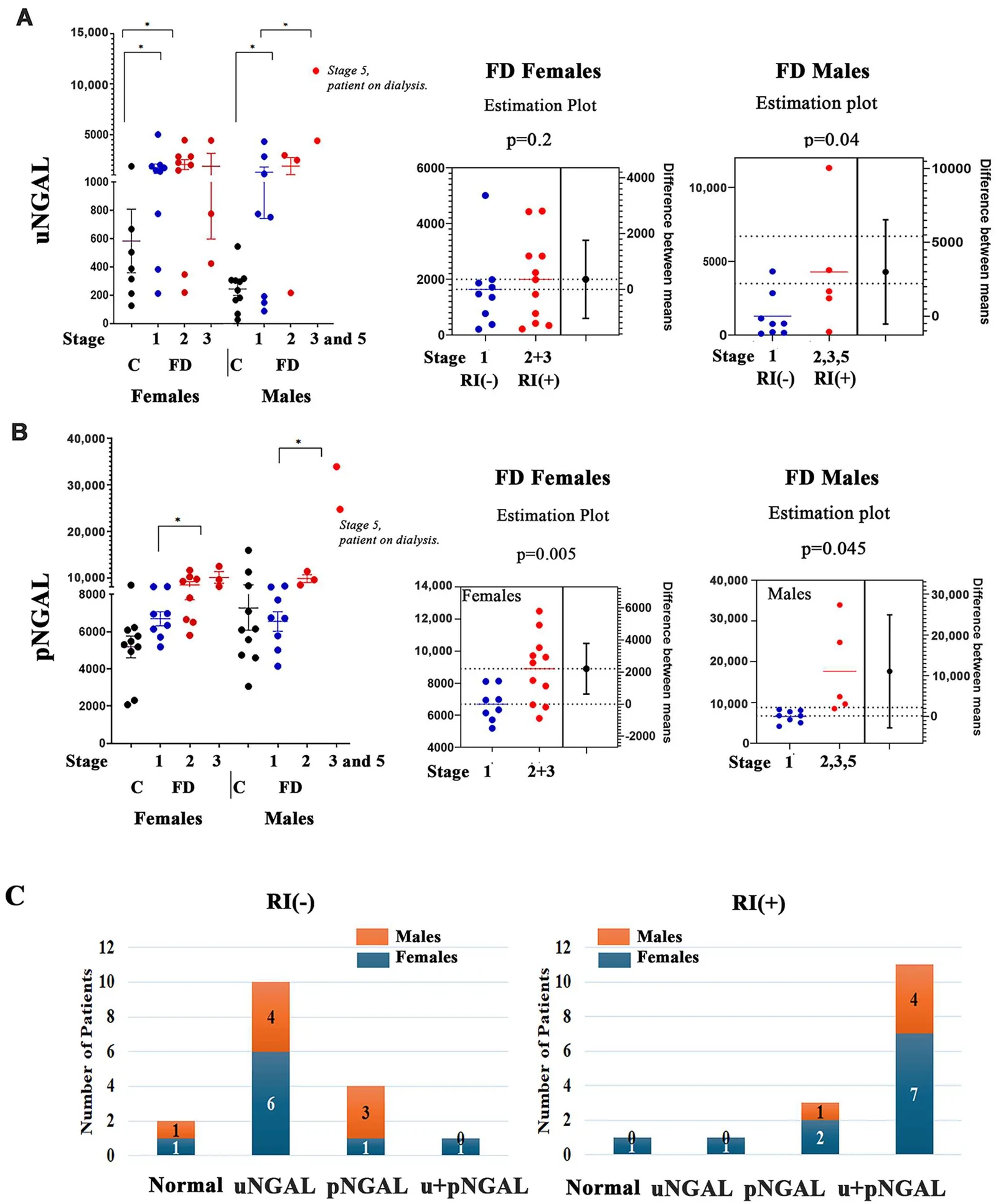
Level of NGAL in comparison to the stage of nephropathy in patients with FD. (**A**) uNGAL levels are compared between healthy controls (C) and patients (FD) with different stages of nephropathy (stages 1, 2, and 3). NGAL (pg/mL) is represented as Mean ± SEM. Ordinary one-way ANOVA summary *p* < 0.0001. Statistical analysis using an unpaired *T*-test between two groups; significantly different: *p* < 0.005. Nonparametric Mann–Whitney U test, one-tailed, *p* = 0.027 control vs. st1 females, *p* = 0.04 control vs. St 2 females, *p* = 0.06 Ct vs. St 1 males, *p* = 0.04 St1 vs. St2, 3, and 5 in FD males. * Present the unpaired *t*-test. Right graphs, estimation plot of uNGAL in male and female FD patients. Individual points represent individual patients. The mean uNGAL level for each group is shown by the horizontal line. The right panel displays the estimated difference in means between Stage 2–3 or 5 and Stage 1, with the 95% confidence interval. (**B**) pNGAL levels are compared between healthy controls (C) and patients (FD) with varying stages of nephropathy (stages 1, 2, and 3, or 5 (one male patient). Right graphs: estimation plot of pNGAL levels in male and female FD patients. Ordinary one-way ANOVA summary *p* < 0.0001. Unpaired *T*-test between two groups; significantly different * *p* < 0.05. Nonparametric Mann–Whitney U test, one-tailed, *p* = 0.02 control vs. St1, females, *p* = 0.01 St1 vs. St2 and 3 females, *p* = 0.008 St1 vs. St 2, 3 and 5, FD males. (**C**) The distribution of cell-specific renal phenotypes in female and male patients is displayed for two groups: Stage 1 (**left panel**) and Stage 2–3 or Stage 5 (**right panel**). Patients were classified based on uNGAL levels using the following cutoffs: uNGAL > 400 and pNGAL > 700. The groups were divided into three categories: elevated uNGAL only, elevated pNGAL only), and elevated uNGAL and pNGAL. The stacked bars represent the number of patients in each category, with separate representations for female and male patients.

NGAL levels increased progressively with advancing nephropathy in FD (Figure 3). Male FD patients with RI+ (Stage 2–3, and 5) disease had significantly higher uNGAL levels compared with those with RI− Stage 1 (estimation plot *p* = 0.04) (Figure 3A). Higher uNGAL levels were observed in male patients with reduced eGFR. Moreover, the male patient on dialysis had the highest uNGAL level. Therefore, because uNGAL is a biomarker of tubular function, we hypothesize that alteration of tubular function is also associated with a significant decline in eGFR in FD males. In contrast, among female patients, elevated uNGAL was detected in the majority of patients across all groups, indicating alteration of tubular function before a detectable decline in eGFR (Figure 3A). As shown in the estimation plot, uNGAL levels were highly variable, and although the mean uNGAL level was higher in female patients with Stage 2–3, the estimated between-group difference was not statistically significant (estimation plot, *p* = 0.20).

Plasma NGAL showed a clear stage-dependent increase in both male and female subjects, with estimated *p*-values of 0.005 and 0.04, respectively (Figure 3B). Female FD patients in stages 2 and 3 had pNGAL levels of approximately 8000–12,000 units, whereas male patients showed higher elevations with progression of nephropathy (Figure 3B). Taken together, pNGAL levels were significantly elevated in advanced nephropathy and increased with stage.

Next, renal phenotypes were assessed according to eGFR-defined disease stage, NGAL level, and sex (Figure 3C). Tubular or glomerular function was defined as uNGAL > 400 as tubular damage and pNGAL > 700 as glomerular damage, or both. In Stage 1 disease, tubular abnormalities (elevated uNGAL) were the most frequently observed phenotype, occurring in 6 of 9 females (66.7%) and 4 of 8 males (50.0%). While only elevated pNGAL were observed in 1 female and 3 males, combined elevation of both biomarkers was observed in one only female FD. In group RI−, one female and one male had normal levels of uNGAL and pNGAL (RI−). In group RI+, the predominant tissue-specific phenotype was combined tubular and glomerular involvement, observed in 7 of 11 females (63.6%) and 4 of 5 males (80.0%). pNGAL elevated were only observed in 2 females and 1 male, whereas uNGAL elevated were only observed in 1 female and no males (Figure 3C). These findings indicate that alteration of tubular function may occur even when glomerular filtration remains normal and may not be adequately captured by conventional eGFR-based kidney disease staging, particularly in female patients.

### 3.3. Negative Linear Correlation Between NGAL and eGFR in FD

**Figure 4:**
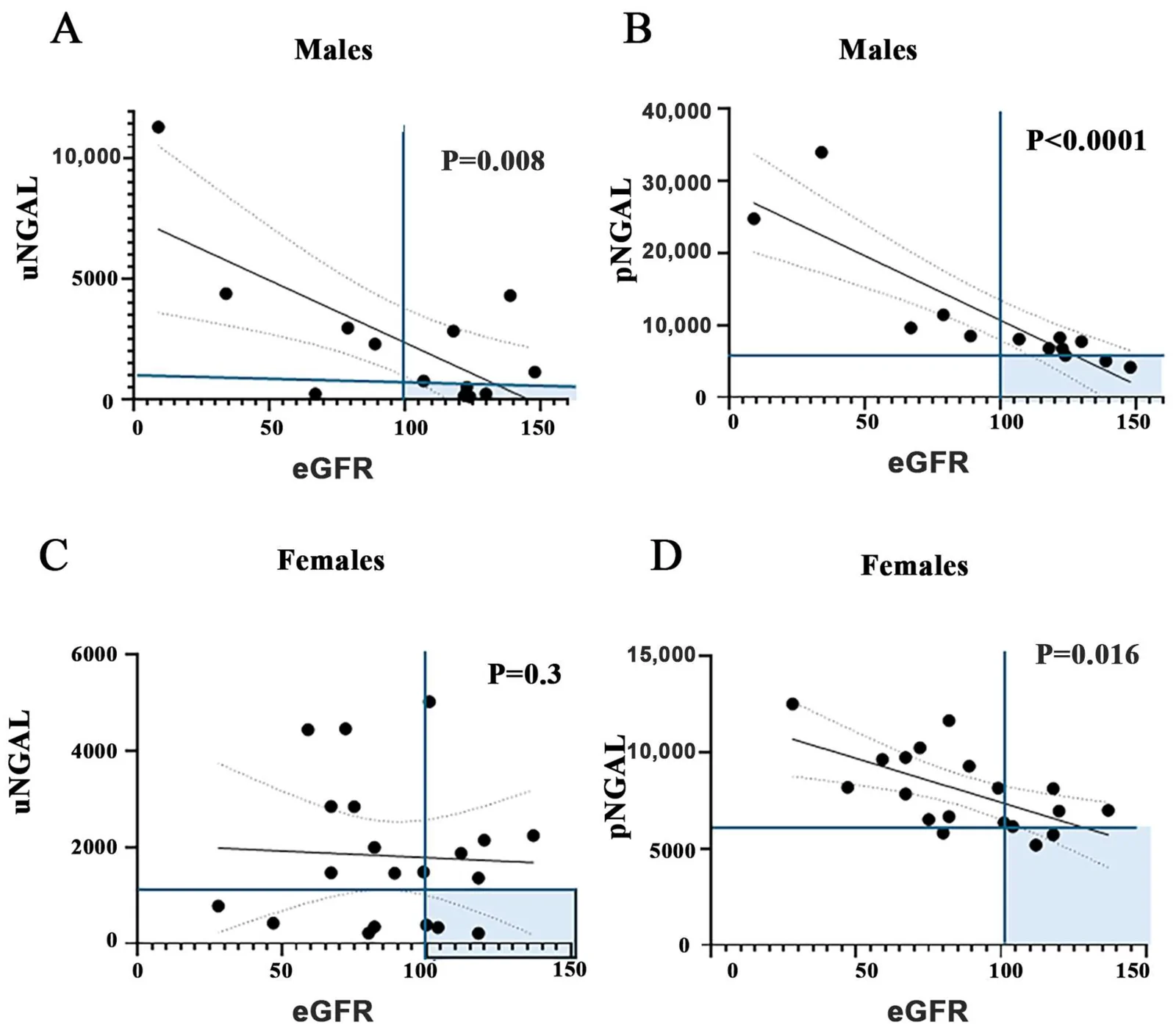
Linear correlation between urinary and plasma NGAL levels and eGFR in male (**A**,**B**) and female (**C**,**D**) patients with FD. The blue rectangle indicates the normal range of NGAL and eGFR levels. Pearson correlation analysis was performed, and *p* values are shown on the graphs.

Correlation analysis in the male FD cohort showed a significant linear correlation between NGAL and eGFR. Urine NGAL (two-tailed Pearson r = −0.695, R^2^ = 0.483, *p* = 0.0083, **) showed a moderate negative correlation with eGFR in male patients (Figure 4A,B). Plasma NGAL (two-tailed Pearson r = −0.864, R^2^ = 0.746, *p* = 0.0001, ****) exhibited the strongest negative correlation with eGFR in the dataset.

In contrast, female patients showed a significant negative correlation between pNGAL and eGFR (Pearson r = −0.528, R^2^ = 0.279, two-tailed *p* = 0.0167), but no correlation between uNGAL and eGFR (Figure 4C,D). These sex-specific findings suggest that in FD females, tubular injury (as reflected by elevated uNGAL) may occur independently of, or prior to, significant impairment of glomerular filtration (as measured by declining eGFR). This pattern supports the possibility of early tubular atrophy in female FD patients before overt glomerular dysfunction becomes apparent.

To better understand the asymptomatic stages of nephropathy in FD that precede the decline in eGFR, we stratified the cohort to isolate patients displaying renal hyperfiltration (higher eGFR). Hyperfiltration was defined using an age- and sex-adjusted threshold. For individuals aged 18–29, the baseline eGFR is set at ≥125 mL/min/1.73 m^2^ for males and ≥120 mL/min/1.73 m^2^ for females. For those aged 30 and above, the threshold is eGFR ≥ 120 mL/min/1.73 m^2^ for males and eGFR ≥ 115 mL/min/1.73 m^2^ for females. During the hyperfiltration phase, no statistically significant differences in either uNGAL or pNGAL levels were observed between the male and female groups with stage 1 kidney involvement.

### 3.4. eGFR and pNGAL, Not uNGAL, Correlate with Age

**Figure 5:**
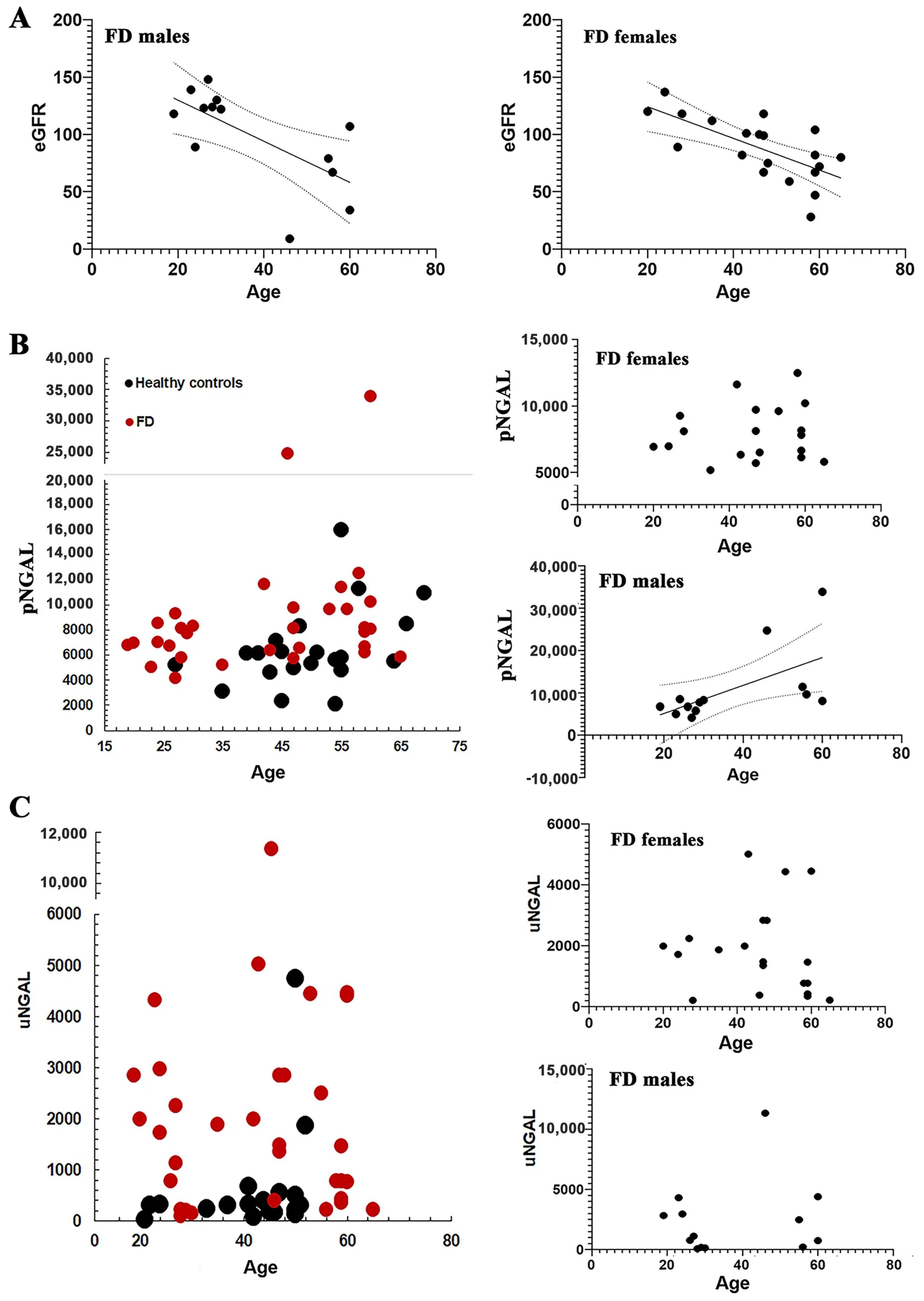
Correlation analysis of eGFR, urinary and plasma NGAL, and age. (**A**) The correlation between eGFR and age in male and female patients with FD. Pearson correlation, *p* = 0.006 in FD males and *p* = 0.0005 in FD females. (**B**) The correlation between pNGAL and age in healthy controls (black circles, Pearson correlation *p* = 0.02) and FD patients (red circles, Pearson correlation *p* = 0.045). The right panel shows the correlation between pNGAL and age in female and male FD patients separately. (**C**) A scatter plot showing the correlation between uNGAL and age in healthy controls (black circles, Pearson correlation *p* = 0.10) and FD patients (red circles, Pearson correlation *p* = 0.46). The right panel displays the correlation between pNGAL and age for female and male FD patients.

Based on previous studies demonstrating an age-related decline in eGFR in the general population and in patients with FD [24,25,26,27,28,29], we hypothesized that increasing age would be associated with lower eGFR in our FD cohort. Moreover, because eGFR was inversely correlated with NGAL (Pearson correlation, two-tailed, Figure 2), we further hypothesized that increasing age would be associated with higher NGAL levels. Age showed a significant inverse correlation with eGFR in the overall FD cohort (r = −0.67, *p* < 0.0001; Table 3; Figure 5A). This association was confirmed after stratification by sex, with significant inverse correlations in female (r = −0.68, *p* = 0.0005) and male (r = −0.67, *p* = 0.006) patients with FD.

Age was positively correlated with pNGAL in healthy controls (r = 0.4, *p* = 0.02) and in the FD cohort (r = 0.3, *p* = 0.045; Figure 5B). Within the FD cohort, this association was observed in male patients (r = 0.59, *p* = 0.015), but not in female patients (r = 0.1, *p* = 0.33). In contrast, uNGAL demonstrated no significant correlation with age in healthy controls or in the overall FD cohort (Table 3 and Figure 5C). No significant association was observed in female patients or male patients. Therefore, these findings indicate that the relationship between age and NGAL levels is more evident for circulating pNGAL than for uNGAL in FD.

### 3.5. Circulating Gb-3/Lyso-Gb-3 and Kidney Involvement in FD Patients

**Figure 6:**
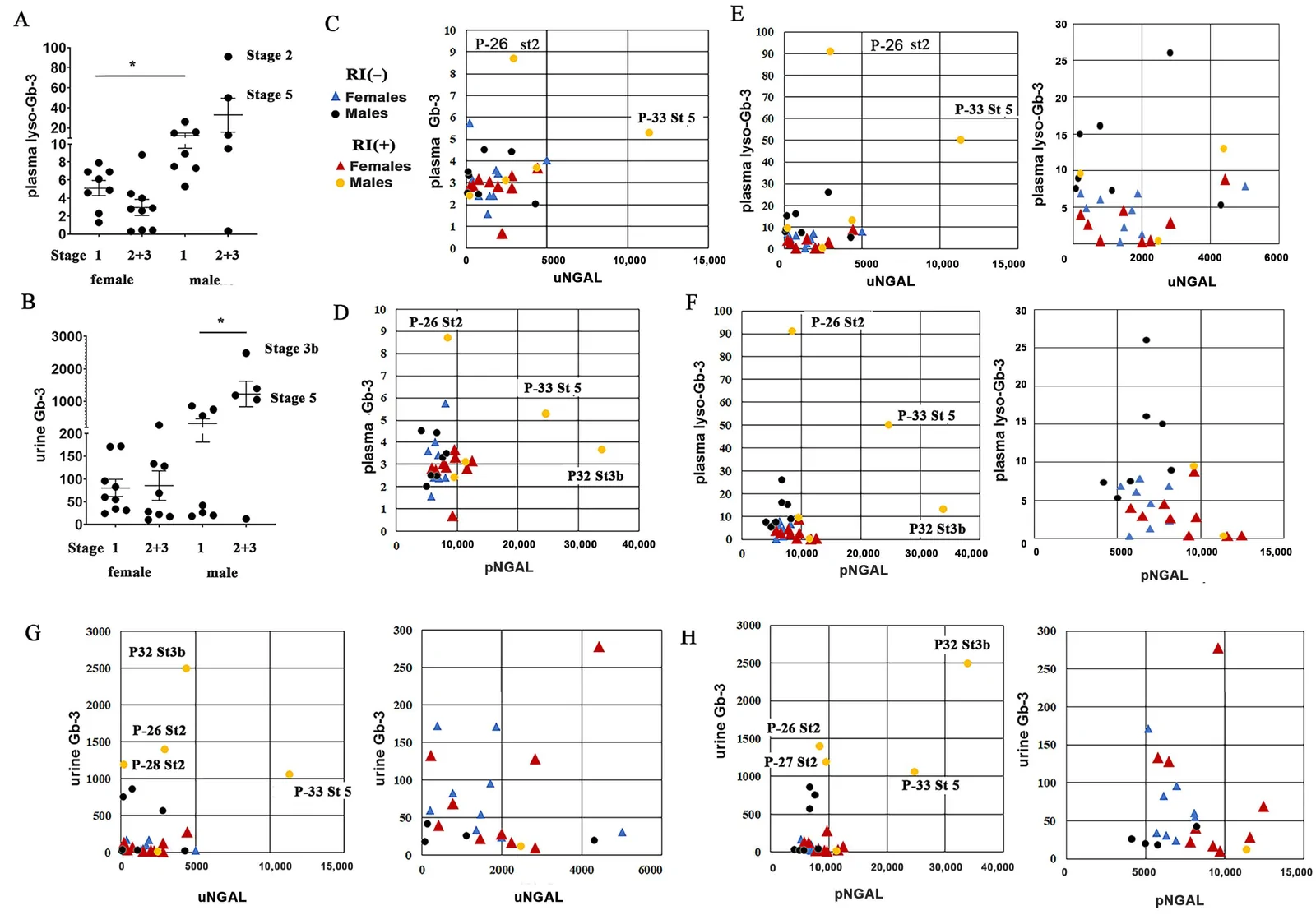
Gb3/lyso-Gb3 and NGAL levels in FD patients. (**A**) Plasma level of lyso-Gb-3 in male and female FD patients without (stage 1) and with (stage 2 and 3) renal involvement. One-way ANOVA demonstrated a significant difference among the group means (F = 4.434, *p* = 0.0125). An unpaired two-tailed Student’s *t*-test demonstrated a significant difference between males and females in Stage 1, and between males and females in Stage 2 + 3 (* *p* = 0.02 and *p* = 0.0309, respectively). (**B**) uGb-3 in male and female FD patients with stage 1 and stage 2 + 3 kidney nephropathy. Kruskal–Wallis test, NS. An unpaired two-tailed Student’s *t*-test demonstrated a significant difference between stage 1 and stages 2 + 3 in FD males (*p* = 0.035). * *p* < 0.05. (**C**,**D**) Correlation between pGb3 and uNGAL (**C**), pGb3 and p NGAL (**D**) in male and female patients with FD. Pearson correlation analysis revealed no significant correlations. (**E**,**F**) The correlation between lyso-Gb-3 and uNGAL (**E**), or pNGAL (**F**). The right panels show a zoomed view. Pearson correlation analysis showed a significant correlation between lyso-Gb-3 and uNGAL (*p* = 0.01). (**G**) The left panel shows the correlation between uGb-3 (maximum value 3000 μg/mmol/creatinine) and uNGAL in FD patients. The left panel shows the same correlation with the y-axis scaled to a maximum uGb-3 concentration of 300 μg/mmol/creatinine (zoomed view). (**H**) The correlation between uGb-3 (maximum 3000 μg/mmol/creatinine) and pNGAL in FD in the left panel. The right panels show a zoomed view of uGb-3, scaled to a maximum of 300 μg/mmol/creatinine. Symbols: blue triangle (females, stage 1); red triangle (females, stage 2 + 3); black circle (male, stage 1); yellow circle (male, Stage 2, 3, and 5).

Gb-3 and lyso-Gb-3 served as key clinical biomarkers for the diagnosis of FD [30]. Lyso-Gb-3 proved to be a highly sensitive biomarker for monitoring disease progression, showing a stronger correlation with disease severity, particularly in male patients. Treatment-naïve patients had elevated levels of both substrates, which decreased shortly after treatment initiation with ERT or chaperone therapy [30,31,32]. In our cohort, plasma Gb-3 showed only slight variation across groups (Table 4). The levels of lyso-Gb-3 were significantly higher and more variable in males compared to females, particularly in patients with FN (Table 4 and Figure 6A). Similarly, urinary Gb-3 levels were elevated in the male patients with advanced nephropathy, including those with stage 3b and stage 5 disease (Table 4 and Figure 6B). These findings indicate that increased plasma and urinary sphingolipid levels were associated with more advanced renal involvement in male patients with FD.

The intracellular deposition of Gb-3 and lyso-Gb-3 takes place in all renal cells. Inclusion Gb-3 in podocytes leads to detachment of the dead podocytes and loss of these cells in urine [33]. While long-lived, non-dividing podocytes accumulate Gb-3 over time, dividing tubular cells exhibit lower levels of Gb-3 deposition. This effect is not a result of less Gb-3 accumulation, but an effect of cell division [33,34]. Therefore, urinary Gb-3 primarily originates from lysosomes in kidney tubular cells, which release the lipid into the urinary collecting system [35,36,37,38]. Given the association of increased sphingolipid levels with more advanced nephropathy in our cohort (Figure 6A,B) and the presence of Gb-3 in urine, mostly a result of renal tubular processes, we hypothesized that higher sphingolipid levels would be associated with elevated NGAL. We therefore performed directional Pearson correlation analyses to evaluate the associations between plasma and urinary sphingolipids and pNGAL and uNGAL (Table 5).

Correlation analysis of pGb-3 and NGAL showed that male patients with advanced stages of nephropathy, particularly in stage 3b and stage 5, had significantly higher pGb-3 and uNGAL levels, but no significant linear correlation was observed between the two biomarkers (Figure 6C,D). In contrast, a significant linear correlation was found between plasma lyso-Gb3 and uNGAL when male and female patients were analyzed together (Figure 6E,F, Table 5). Subgroup analysis by sex did not confirm this association, likely due to reduced sample sizes and decreased statistical power for Pearson correlation. Additionally, a significant linear correlation was observed between uGb-3 and plasma/urine NGAL (Figure 6G,H, and Table 5). Analysis by sex confirmed a significant correlation between pNGAL and urine Gb-3 in males, which was also driven by advanced nephropathy. Overall, these findings suggest that increased sphingolipid levels are associated with more advanced nephropathy and higher NGAL levels in patients with FD. Overall, these findings suggest that the uGb-3–pNGAL association was particularly evident in male patients.

### 3.6. Urine NGAL and Creatinine

**Figure 7:**
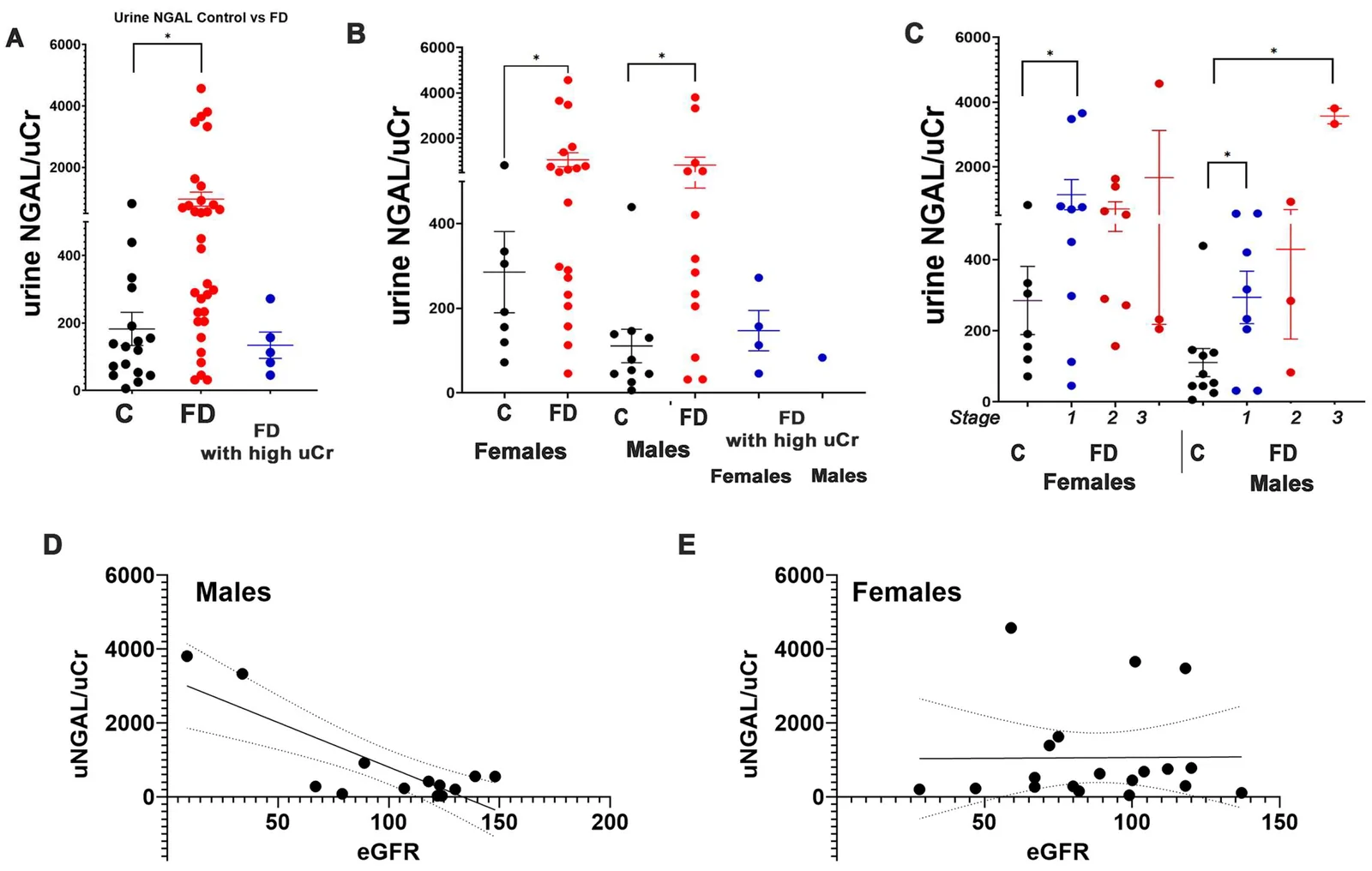
uNGAL with normalization to urinary creatinine (uCr). (**A**) uNGAL/uCr ratio levels are compared between healthy controls and patients with FD. Statistical analysis using an unpaired *T*-test of the cohorts demonstrated a significant difference between the control and the FD cohorts. * *p* < 0.05 *T*-test. (**B**) Sex-specific analysis of the uNGAL/uCr ratio. Samples from FD patients with elevated uCr concentrations are highlighted separately in the blue column to assess the potential impact of creatinine normalization. Student’s *t*-test (* *p* < 0.05). (**C**) uNGAL/uCr ratio in comparison to the stage of nephropathy in patients with FD. (* *p* < 0.05). (**D**,**E**) Correlation between the uNGAL/uCr ratio and eGFR in male (**D**) and female (**E**) patients with FD. Pearson correlation analysis revealed a significant association in males (*p* < 0.01) but not in females.

While the utility of normalizing urinary biomarkers to urinary or plasma creatinine concentration (Cr) remains debated, particularly regarding its impact on the interpretation of kidney function, we evaluated differences in uNGAL with and without normalization to urinary creatinine (uCr) [39,40]. In our analysis, there were no statistically significant differences in uCr levels between healthy controls and patients; the mean uCr was 2.6 ± 0.479 mg/dL in controls versus 5.2 ± 1.35 mg/dL in FD. Only five FD patients showed elevated uCr levels: two females with normal eGFR (uCr level in P2: 15.2 mg/dL; P5: 32.2 mg/dL), two females with reduced eGFR (P21: 10.4 mg/dL; P23: 12.7 mg/dL), and one male patient with FN stage 2 (P28: 29.8 mg/dL). Consistent with previous uNGAL results, the uNGAL/uCr ratio was significantly higher in patients with FD (Figure 7A). Furthermore, female and male patients exhibited a significantly higher uNGAL/uCr ratio than healthy controls (Figure 7B). Male FD patients had the highest uNGAL/uCr ratio in stage 3b and stage 5 nephropathy. However, females showed an increased uNGAL/uCr ratio regardless of stages of kidney involvement (Figure 7C). Similar to unadjusted uNGAL levels, the uNGAL/uCr ratio showed a significant inverse correlation with eGFR in males but not in female patients with FD (Figure 7D,E).

This result indicates that the uCr level did not differ significantly between control and FD patients. As a result, normalization did not enhance the identification of FN patients in our cohort. Therefore, measuring uNGAL alone may be sufficient to assess tubular function in FD, particularly if uCr levels remain within a comparable range across study groups. However, the relatively small sample size limits definitive conclusions regarding the utility of creatinine normalization. Larger studies are necessary to determine whether uNGAL/uCr offers diagnostic value over uNGAL concentrations for monitoring kidney disease progression in FD.

### 3.7. Plasma NGAL Associated with Proteinuria in FD Patients

**Figure 8:**
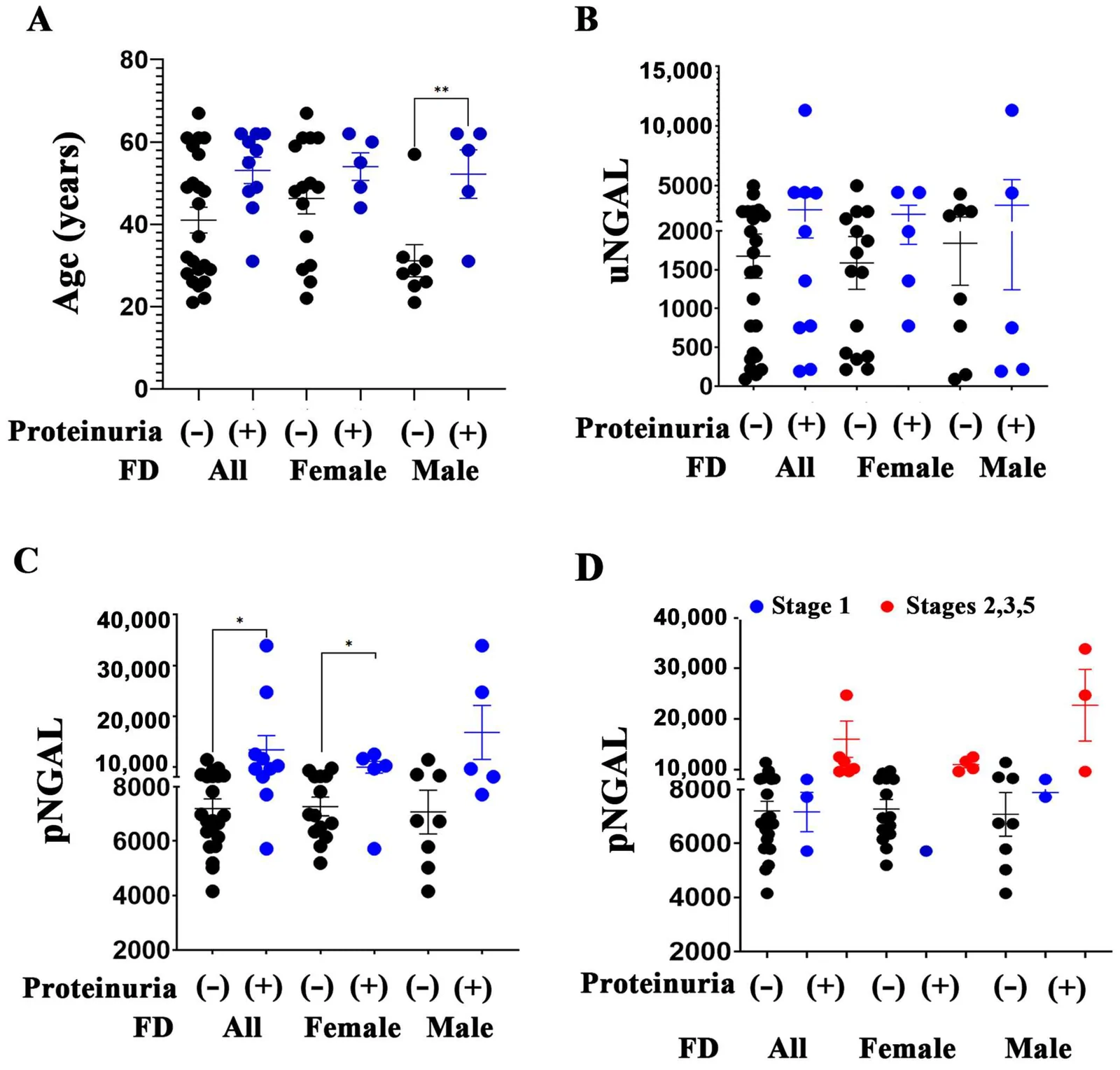
Association of proteinuria with age and NGAL levels in patients with FD. (**A**) Patients’ ages at diagnosis of proteinuria. Comparison of age in FD males with and without proteinuria: *p* = 0.004. (**B**) uNGAL levels in FD patients in the absence (−) or presence (+) of proteinuria in the entire cohort and after sex stratification. (**C**) pNGAL levels in FD patients stratified in the absence (−) or presence (+) of proteinuria and grouped by sex. pNGAL was significantly higher in FD patients with proteinuria overall and among female patients. (**D**) pNGAL concentrations stratified by kidney disease stage and proteinuria status. Blue symbols represent Stage 1 patients, whereas red symbols represent patients with Stages 2, 3, or 5. Each dot represents an individual patient. *T*-test statistical analysis represents the mean ± SEM. * *p* < 0.05; ** *p* < 0.01.

To determine if circulating NGAL was associated with proteinuria in FD, plasma and urinary NGAL were compared between patients with and without proteinuria. In our cohort, proteinuria was documented in 10 FD patients based on Urine Albumin-to-Creatinine Ratio (UACR) and Urine Protein-to-Creatinine Ratio (UPCR) values extracted from clinical charts. Eight patients had elevated UACR, ranging from 33 to 1286 mg/g creatinine (reference <30 mg/g), with the highest values of 1286 and 801 mg/g indicating substantial albuminuria. One patient had a markedly elevated UPCR of 1237 mg/g creatinine (reference <200 mg/g). An additional, one patient had 3+ proteinuria by urine dipstick. Age was comparable between proteinuria-negative and proteinuria-positive groups in the overall FD cohort and among females. However, in our cohort, male patients with proteinuria were significantly older than patients without proteinuria (*p* < 0.01) (Figure 8A).

pNGAL, but not uNGAL, was significantly elevated in patients with proteinuria (Figure 8B,C). When stratified by sex, this difference remained significant in female patients, whereas in male patients it did not reach statistical significance. Notably, several male patients with proteinuria showed markedly elevated pNGAL levels. Because pNGAL showed a stronger relationship with eGFR, whereas proteinuria in this cohort was primarily defined by increased urinary albumin/protein excretion, we further evaluated pNGAL according to kidney disease stage. Patients with Stage 2, 3, or 5 disease and proteinuria generally had higher pNGAL levels than those without proteinuria or stage 1 with proteinuria (Figure 8D). Together, these findings indicate that pNGAL levels were higher in patients with proteinuria and in those with more advanced kidney disease stages.

## 4. Discussion

Nephropathy is one of the leading causes of morbidity and mortality in FD. Diagnostic clinical biomarkers such as eGFR, serum creatinine, and cystatin C are not sensitive for detecting early renal involvement, particularly the tubular injury that may precede overt glomerular dysfunction and proteinuria in FD [41,42]. This study evaluated the role of plasma and urinary NGAL as noninvasive biomarkers for assessing kidney disease progression in patients with FD.

NGAL is a sensitive clinical biomarker for detecting early kidney pathology, particularly tubular injury, although its clinical value across different renal diseases remains under investigation [16,17,23]. Moreover, urinary NGAL has been suggested as a non-traditional kidney biomarker in FD [43,44]. To our knowledge, this is the first study to evaluate plasma and urinary NGAL levels in patients with FD and to examine their associations with sex, age, eGFR, Gb3, and lyso-Gb3. The results demonstrate that urinary and plasma NGAL are significantly elevated in patients with FD. Moreover, uNGAL was elevated in both male and female FD patients in comparing with healthy controls, even in those classified as Stage 1 (eGFR > 90 mL/min/1.73 m^2^) with minimal or no clinical evidence of nephropathy. This finding supports the notion that subclinical tubular injury occurs early in the disease course, which is consistent with evidence of Gb3 accumulation in tubular epithelial cells and podocytes long before declines in eGFR become apparent [33,45,46].

A key observation was the elevation of NGAL levels with advancing stages of nephropathy. In male patients, urine and plasma NGAL increased significantly with declining eGFR, reaching the highest values in Stage 3b and 5, including the patient on dialysis. This data aligns with the more severe, rapidly progressive phenotype observed in classically affected hemizygous males. In contrast, female patients exhibited significantly elevated urine NGAL across all stages, regardless of eGFR levels. Plasma NGAL in FD females, however, significantly increased in Stages 2 and 3. Therefore, tubular damage (as reflected by urine NGAL) may manifest earlier and may be less dependent on global glomerular filtration rate in females.

Correlation analyses further strengthened these findings. In male patients, both urine and plasma NGAL showed significant negative linear correlations with eGFR, with pNGAL demonstrating the strongest association (r = −0.660, *p* < 0.0001). This suggests that pNGAL effectively reflects eGFR and reduced proximal tubular reabsorption capacity in advanced disease. In females, only pNGAL was significantly correlated with eGFR, whereas uNGAL was not. This dissociation supports the hypothesis that tubular injury can occur as an early, somewhat independent event in female FD patients, potentially driven by direct Gb3/lyso-Gb3-mediated cellular stress and inflammation in tubular epithelium before significant glomerular involvement. These findings are consistent with previous reports that kidney pathology in female FD patients may progress even while clinical standard urinary biomarkers remain within normal ranges [33,47].

Although the need to adjust eGFR thresholds for age remains a long-standing debate [48], we found that age was significantly correlated with eGFR in male and female patients, underscoring the clinical relevance of age as an important factor in predicting kidney function in this FD cohort. Plasma NGAL tends to gradually increase with age in the general population. Moreover, individuals aged 45 and older generally exhibit higher baseline NGAL levels than younger populations [48]. In our study, pNGAL levels increased with age in both controls and male patients with FD, whereas uNGAL levels correlated with age only in male patients with FD. These findings highlight that aging affects kidney and cardiac functions in FD. Additionally, plasma NGAL may reflect the cumulative disease burden over time. Furthermore, these results emphasize the need for future research to investigate the mechanisms underlying NGAL function to predict disease progression.

Fabry-specific biomarkers Gb3 and lyso-Gb3 showed moderate and more prominent correlations with renal function parameters in the FD male cohort. In contrast to Gb3-/lyso-Gb-3, NGAL demonstrated clearer stage-dependent changes and stronger correlation with eGFR. This is clinically relevant because patients receiving ERT or CT therapies often see reductions in circulating lyso-Gb3 levels without necessarily halting ongoing kidney manifestations or fibrosis [49,50]. However, NGAL, as a biomarker, may directly indicate active renal cellular function and the progression of nephropathy.

Mechanistically, the elevation of NGAL in FD nephropathy may increase through several cellular pathways. The accumulation of Gb3 and lyso-Gb3 disrupts lysosomal function and impairs autophagic flux. Consequently, it triggers excessive generation of reactive oxygen species (oxidative stress) and activates the NF-κB and Notch1 pathways, which serve as a major inflammatory bridge that may directly intersect with and amplify the EGFR/HIF-1α/NGAL axis [21,51,52,53]. In the context of tubular function in FD, results indicate that increased urinary NGAL excretion is driven by a synergistic double hit: NGAL synthesis by stressed distal tubular cells while simultaneously disrupting the reabsorption of filtered NGAL in the damaged proximal tubule. Elevated plasma NGAL may result from activated neutrophils/monocytes, leading to chronic inflammation and reduced renal clearance due to glomerular and tubular dysfunction.

NGAL is a promising complementary biomarker to traditional renal function tests in FD. Urinary NGAL appears useful for detecting early tubular injury and potentially interstitial fibrosis, while plasma NGAL may provide complementary information related to overall renal dysfunction and glomerular function. Including NGAL measurements alongside standard biomarkers for monitoring kidney function in FD could help identify renal involvement earlier, facilitate timely initiation or optimization of disease-specific therapies, and monitor treatment response. Moreover, multiple cell types and cellular pathways contribute to the pathogenesis of Fabry nephropathy. Other biomarkers of renal injury, including KIM-1, clusterin, bikunin, or YKL40 have been investigated as complementary markers of tubular injury, fibrosis, and renal pathology [8,54]. The development of a screening panel incorporating biomarkers with complementary cell specificity may provide a more comprehensive assessment of renal involvement and disease progression in FD [7,8,9,55,56].

Limitations of the present study include the modest sample size, particularly when stratified by nephropathy stage and further by sex-specific groups. Assessment of NGAL as a single biomarker provides a relatively narrow view of the cellular mechanisms underlying kidney impairment in FD. Future studies should therefore expand the evaluation to additional tubular and inflammatory biomarkers to allow a more comprehensive understanding of renal involvement. Larger longitudinal studies are also needed to confirm the clinical utility of NGAL and to explore its potential role in guiding personalized management of Fabry nephropathy.

## 5. Conclusions

This study demonstrates that plasma and urinary NGAL levels are significantly elevated in patients with FD, including those with early-stage nephropathy and preserved eGFR. Urinary NGAL reflects renal tubular injury in female and male patients, consistent with its established role as a tubular injury marker, while plasma NGAL shows a stronger association with declining glomerular function, particularly in males. Together, these findings suggest that NGAL may serve as a valuable complementary biomarker for detecting tubular injury alongside conventional measures of glomerular function, providing a more comprehensive assessment of renal involvement in Fabry disease. Prospective longitudinal studies in a larger cohort are warranted to determine whether NGAL can further predict renal disease progression and establish its utility for monitoring kidney function and treatment response in Fabry disease.

## Figures and Tables

**Table 1:** Demographics, clinical, and diagnostic characteristics of patients with FD. The study comprised 33 patients with FD, of whom 13 were male and 20 were female. The mean age for males was 38 ± 15 years, and for females, it was 46 ± 13 years. FD patients with kidney Stage 1 included 9 females and 8 males. Patients with FD nephropathy (Stages 2 and 3) included 11 females and 4 males. One additional male patient with FD had Stage 5 kidney disease and was receiving dialysis. One patient with stage 1 was treatment-naïve (*), and two patients were pseudo-naïve (&). Abbreviations: ERT, enzyme replacement therapy; CT, chaperone therapy; SRT, substrate reduction therapy; CTR, in clinical trial; pGb-3, plasma Gb-3; pLyso-Gb-3, plasma lyso-Gb-3; uGb-3, urinary Gb-3.

ID	Age	Sex	FN Stage	Cardio Yes/No	Therapy	eGFR	Cystatin-C	pGb-3	pLyso-Gb-3	uGb-3	GLA
1	60	F	1	Yes	&	104	N/A	2.41	6.1	83	c.830G > A
2	24	F	1	No	CT	137	0.79	2.4	4.6	96	c.730 G > A
3	21	F	1	No	ERT	120	0.73	3.42	1.3	24	c.984del
4	35	F	1	No	*	112	0.74	3.59	6.9	171	c.1072_1074del
5	48	F	1	No	ERT	99	0.82	2.42	2.3	55	c.668G > A
6	28	F	1	No	ERT	118	0.71	5.74	6.9	60	c.146G > C
7	43	F	1	No	SRT	101	0.82	4.02	7.9	31	c.901 C > T
8	48	F	1	Yes	ERT	118	N/A	1.57	<0.30	<34	c.1088G > A
9	47	F	1	No	CTR	100	0.82	3.17	4.9	172	c.1057_1058del
10	26	M	1	Yes	ERT	123	0.92	2.48	16	856	c.830G > A
11	23	M	1	Yes	ERT	139	N/A	2.02	5.3	20	c.974G > A
12	29	M	1	Yes	ERT	124	0.97	2.52	7.5	<18	c.974G > A
13	30	M	1	No	ERT	130	0.82	3.31	15	754	c.718_719delAA
14	30	M	1	Yes	&	122	0.84	3.48	8.9	42	Mosaic 422C > T
15	20	M	1	No	ERT	118	0.94	4.42	26	566	c.146G > C
16	27	M	1	No	ERT	148	0.66	4.51	7.3	26	c.400T > G
17	61	M	1	No	CT	107	0.77	1.55	0.55	<20	c.122C > T
18	65	F	2	Yes	ERT	80	0.97	2.83	4	<133	c.974G > A
19	49	F	2	Yes	ERT	75	0.94	2.76	2.9	128	c.718_719delAA
20	27	F	2	No	SRT	89	0.97	0.68	0.45	<17	c718_719del AA
21	47	F	2	Yes	ERT	67	1.11	3.3	2.8	10	c.680G > A
22	60	F	2	Yes	ERT	67	1.07	3.04	4.5	<22	c.146 G > C
23	42	F	2	Yes	ERT	82	1.32	2.84	<0.30	28	c.644A > G
24	61	F	2	Yes	ERT	72	1.01	3.51	8.4	419	c.45del
25	59	F	2	No	ERT	84	0.9	2.86	4.8	29	c. 982G > C
26	24	M	2	No	CTR	89	0.95	8.69	91	1393	c.602C > T
27	56	M	2	Yes	ERT	67	1.14	2.41	9.5	1186	c.620A > G
28	56	M	2	No	CT	79	1.01	3.09	0.34	12	c.550G > A
29	59	F	3a	Yes	ERT	47	1.39	2.88	2.6	>40	c.400T > G
30	54	F	3a	Yes	ERT	59	1.19	3.67	8.8	278	c.984DelG/EX6
31	58	F	3b	Yes	No	28	2	3.17	0.43	69	C.886A > G
32	60	M	3b	Yes	ERT	34	2.2	3.67	13	2489	c.717_718del Frameshift
33	47	M	5	No	ERT	9	5.12	5.19	50	1055	c.602 C > T

**Table 2:** Clinical nephropathy staging. The table summarizes the clinical classification of kidney involvement in patients with FD. Nephropathy stages were assigned according to the patients’ clinical diagnoses and staging documented in their medical records. The eGFR and cystatin C values shown in the table represent the ranges observed among patients within each clinically assigned nephropathy stage and are provided for descriptive characterization of kidney function. The table summarizes FD-specific biochemical and genetic characteristics, including α-galactosidase A (α-Gal A) activity, GLA mutations, and substrate accumulation (Gb3 and lyso-Gb3).

	Kidney Involvement in Patients with FD				Age-Matched Controls
Clinical Parameter (s)	Stage 1	Stage 2	Stage 3a	Stage 3b	
**Kidney damage**	Normal	Mild Reduction	Moderate Reduction	Severe Reduction	Normal
**eGFR** mL/min/1.73 m^2^	>90	60–89	45–59	30–44	
**Cystatin-C**	0.5–0.9	1.0–1.2	1.3–1.5	1.4–1.9	0.5–0.9
**FD biochemical markers**					
**α–Gal A**	Deficiency	Deficiency	Deficiency	Deficiency	Normal
**GLA mutations**	Yes	Yes	Yes	Yes	No
**Gb-3**	Yes	Yes	Yes	Yes	No
**Lyso-Gb-3**	Yes	Yes	Yes	Yes	No

**Table 3:** Correlation of age with eGFR, plasma NGAL (pNGAL), and urinary NGAL (uNGAL) in control and FD groups. One-tailed *p* values were used for prespecified directional hypotheses based on prior biological and clinical evidence, specifically an inverse association between age and eGFR and a positive association between age and NGAL. Significance symbols: **** *p* < 0.0001; *** *p* < 0.001; ** *p* < 0.01; * *p* < 0.05; ns = not significant.

Age vs.		R Squared	*p* (One-Tailed)	*p* Value
**eGFR**	FD female + male	0.4	<0.0001	****
	female	0.4	0.0005	***
	male	0.4	0.006	**
**pNGAL**	control	0.1	0.02	*
	FD female + male	0.09	0.045	*
	FD female	0.01	0.33	ns
	FD male	0.35	0.015	*
**uNGAL**	control	0.09	0.10	ns
	FD female + male	0.0002	0.46	ns
	FD female	0.008	0.35	ns
	FD male	0.02	0.3	ns

**Table 4:** Distribution of glycosphingolipid biomarkers (Gb-3 and Lyso-Gb-3) in plasma and urine according to sex and FN status.

	Females		Males	
	RI (−)	RI (+)	RI (−)	RI (+)
**Plasma Gb-3**				
**Mean**	3.2	2.8	3.3	4.6
**Minimum**	1.6	0.7	2.0	2.4
**Maximum**	5.7	3.7	4.5	8.7
**Std. Deviation**	1.2	0.8	1.0	2.5
**Plasma lyso-Gb-3**				
**Mean**	5.1	3.0	12.3	32.8
**Minimum**	1.3	0.3	5.3	0.3
**Maximum**	7.9	8.8	26.0	91.0
**Std. Deviation**	2.3	2.7	7.3	37.7
**Urine Gb-3**				
**Mean**	80.7	85.6	326.0	1227.0
**Minimum**	24.0	10.0	18.0	12.0
**Maximum**	172.0	278.0	856.0	2489.0
**Std. Deviation**	56.7	91.9	383.0	884.0

**Table 5:** The Pearson linear correlation between uGb-3 and plasma Lyso-Gb3 and NGAL. One-tailed *p* values were used for these directional analyses. Only correlations with *p* ≤ 0.05 are indicated as significant. * *p* < 0.05, ** *p* < 0.01, **** *p* < 0.0001.

	Pearson r	95% Confidence Interval	R Squared	*p* (One-Tailed)	*p* Value
**Plasma Lyso-Gb3**					
urine NGAL (male + female)	0.4174	0.06726 to 0.6760	0.1742	0.0109	*
urine NGAL (male)	0.4180	−0.2051 to 0.8000	0.1748	0.08	No
urine NGAL (female)	0.3114	−0.1819 to 0.6795	0.09696	0.1042	No
plasma NGAL (male + female)	0.2326	−0.1393 to 0.5470	0.05410	0.1081	No
plasma NGAL (male)	0.1415	−0.3267 to 0.7642	0.02001	0.1542	No
plasma NGAL (female)	−0.3758	−0.7254 to 0.1280	0.1412	0.0686	No
**Urine Gb3**					
urine NGAL (male + female)	0.3241	−0.04090 to 0.6128	0.1051	0.0403	*
urine NGAL (male)	0.3070	−0.3240 to 0.7489	0.09422	0.1659	No
urine NGAL (female)	0.1378	−0.3517 to 0.5681	0.01899	0.2928	No
plasma NGAL (male + female)	0.7115	0.4722 to 0.8531	0.5062	<0.0001	****
plasma NGAL (male)	0.7573	0.3243 to 0.9279	0.5735	0.0022	**
plasma NGAL (female)	−0.1492	−0.5877 to 0.3570	0.02228	0.2838	No

## Data Availability

The original contributions presented in this study are included in the article. Further inquiries can be directed to the corresponding author.

## Abbreviations

The following abbreviations are used in this manuscript:

FD	Fabry disease
FN	Fabry nephropathy
NGAL	Neutrophil Gelatinase-Associated Lipocalin
eGFR	estimated Glomerular Filtration Rate
ERT	Enzyme Replacement Therapy
NF-κB	Nuclear Factor kappa-light-chain-enhancer of activated B cells
Notch1	Notch receptor 1
EGFR	Epidermal Growth Factor Receptor
HIF-1α	Hypoxia-Inducible Factor 1-alpha
Gb-3	Globotriaosylceramide
Lyso-Gb-3	Globotriaosylsphingosine
pNGAL	Plasma NGAL
uNGAL	Urine NGAL
